# On the Effects of Reactive Oxygen Species and Nitric Oxide on Red Blood Cell Deformability

**DOI:** 10.3389/fphys.2018.00332

**Published:** 2018-05-11

**Authors:** Lukas Diederich, Tatsiana Suvorava, Roberto Sansone, T. C. Stevenson Keller, Frederik Barbarino, Thomas R. Sutton, Christian M. Kramer, Wiebke Lückstädt, Brant E. Isakson, Holger Gohlke, Martin Feelisch, Malte Kelm, Miriam M. Cortese-Krott

**Affiliations:** ^1^Cardiovascular Research Laboratory, Division of Cardiology, Pneumology and Vascular Medicine, Medical Faculty, Heinrich-Heine-University Düsseldorf, Düsseldorf, Germany; ^2^Department of Molecular Physiology and Biological Physics, Robert M. Berne Cardiovascular Research Center, University of Virginia, Charlottesville, VA, United States; ^3^Clinical & Experimental Sciences, Faculty of Medicine, University of Southampton, Southampton, United Kingdom; ^4^Faculty of Mathematics and Natural Sciences, Institute for Pharmaceutical and Medicinal Chemistry, Heinrich-Heine-University Düsseldorf, Düsseldorf, Germany; ^5^Medical Faculty, Cardiovascular Research Institute Düsseldorf, Heinrich-Heine-University Düsseldorf, Düsseldorf, Germany

**Keywords:** erythrocytes, thiols, non-canonical function, mechanotransduction, RBC deformability, nitric oxide synthase, reactive oxygen species

## Abstract

The main function of red blood cells (RBCs) is the transport of respiratory gases along the vascular tree. To fulfill their task, RBCs are able to elastically deform in response to mechanical forces and, pass through the narrow vessels of the microcirculation. Decreased RBC deformability was observed in pathological conditions linked to increased oxidative stress or decreased nitric oxide (NO) bioavailability, like hypertension. Treatments with oxidants and with NO were shown to affect RBC deformability *ex vivo*, but the mechanisms underpinning these effects are unknown. In this study we investigate whether changes in intracellular redox status/oxidative stress or nitrosation reactions induced by reactive oxygen species (ROS) or NO may affect RBC deformability. In a case-control study comparing RBCs from healthy and hypertensive participants, we found that RBC deformability was decreased, and levels of ROS were increased in RBCs from hypertensive patients as compared to RBCs from aged-matched healthy controls, while NO levels in RBCs were not significantly different. To study the effects of oxidants on RBC redox state and deformability, RBCs from healthy volunteers were treated with increasing concentrations of *tert*-butylhydroperoxide (*t-*BuOOH). We found that high concentrations of *t*-BuOOH (≥ 1 mM) significantly decreased the GSH/GSSG ratio in RBCs, decreased RBC deformability and increased blood bulk viscosity. Moreover, RBCs from Nrf2 knockout (KO) mice, a strain genetically deficient in a number of antioxidant/reducing enzymes, were more susceptible to *t*-BuOOH-induced impairment in RBC deformability as compared to wild type (WT) mice. To study the role of NO in RBC deformability we treated RBC suspensions from human volunteers with NO donors and nitrosothiols and analyzed deformability of RBCs from mice lacking the endothelial NO synthase (eNOS). We found that NO donors induced S-nitrosation of the cytoskeletal protein spectrin, but did not affect human RBC deformability or blood bulk viscosity; moreover, under unstressed conditions RBCs from eNOS KO mice showed fully preserved RBC deformability as compared to WT mice. Pre-treatment of human RBCs with nitrosothiols rescued *t*-BuOOH-mediated loss of RBC deformability. Taken together, these findings suggest that NO does not affect RBC deformability *per se*, but preserves RBC deformability in conditions of oxidative stress.

## Introduction

The biochemical, biophysical, and mechanical properties of RBCs, as well as their structural characteristics are optimized for their function. RBCs carry a very high (supersaturated) concentration of hemoglobin (equivalent to 10 mM heme), which is kept in the reduced Fe^2+^/oxygen binding state by a battery of antioxidant and reducing enzymes (Kuhn et al., 2017). In addition, the peculiar cytoskeleton, which is mainly composed of hexagonal aligned units of spectrin, confers stability, flexibility, and elasticity of the cells. The distribution and biophysical characteristics of the cytoskeletal proteins (mainly spectrin) are responsible for their typical biconcave “donut-like” shape. RBC shape and deformability allow the cells to dynamically adapt to changes in hydrodynamic forces along the vascular tree, and to squeeze through capillaries smaller than their own diameter at rest (Kuhn et al., 2017).

RBC deformability was found to be decreased in several disease states associated with oxidative stress and endothelial dysfunction and/or impaired nitric oxide (NO) bioavailability, such as hypertension and diabetes (Cicco and Pirrelli, 1999; Cicco et al., 1999a,b, 2001; Turchetti et al., 1999; Vetrugno et al., 2004; Radosinska and Vrbjar, 2016; Lee et al., 2017). Interestingly, a linear relationship between deformability and RBC oxidative stress has been documented in studies involving sickle cell disease patients (Barodka et al., 2014a). In disease states, reactive oxygen species (ROS)-mediated damage of RBC membrane components is thought to increase erythrocyte membrane rigidity and fragility, resulting in intravascular hemolysis, release of hemoglobin into the plasma, and systemic NO scavenging. In spite of the clinical significance of these phenomena the biological chemistry and biochemistry of the processes that control physiological RBC deformability in health and disease, and the underlying signaling pathways remain poorly characterized.

There is compelling evidence that treatment of RBCs with thiol-reactive molecules such as diamide or oxidants such as peroxides not only strongly impair RBC deformability (Fischer et al., 1978; Corry et al., 1980), but also change RBC shape (Becker et al., 1986; Mcgough and Josephs, 1990), suggesting an important role of intracellular redox status in control of RBC deformability and structural characteristics. In contrast, treatment with NO donors was shown to improve RBC deformability (Bor-Kucukatay et al., 2003; Grau et al., 2013; Riccio et al., 2015), while treatment with nitric oxide synthase (NOS) inhibitors impaired RBC deformability (Bor-Kucukatay et al., 2003; Grau et al., 2013, 2015). *S*-nitrosation of intracellular proteins such as hemoglobin or spectrin (Grau et al., 2015) and/or activation of soluble guanylate cyclase (sGC) (Bor-Kucukatay et al., 2003) have all been proposed to be involved in these effects. However, these findings are not without controversy. Recent studies have shown that treatment of human RBCs with the NO donors DEA/NO, sodium nitroprusside, and NO synthase (NOS) substrate L-arginine did not improve deformability of RBCs (Barodka et al., 2014b; Belanger et al., 2015). Similarly, neither NOS inhibition nor inhibition of sGC in RBCs affect their deformability (Barodka et al., 2014b; Cortese-Krott et al., 2017). The reasons for these discrepancies are unknown.

In this work, we investigated whether and how changes in intracellular redox status of RBCs, NO, and nitrosation reactions affect RBC deformability. According to previous studies referred to above (Sandhagen et al., 1990; Vaya et al., 1992; Cicco and Pirrelli, 1999; Cicco et al., 1999a,b, 2001; Radosinska and Vrbjar, 2016), we found that hypertensive patients show decreased RBC deformability; interestingly, this was accompanied by an increase in intracellular ROS levels, with unchanged intracellular NO levels. In *ex vivo* experiments carried out with isolated RBCs, we found that changes in intracellular redox status provoked by an oxidant challenge with high concentrations of *t*-BuOOH decreases RBC deformability and increases blood viscosity. Although treatment with NO donors did not significantly affect RBC deformability *per se*, it protected RBCs from adverse changes induced by oxidants. Preserving the antioxidant capacity of RBCs would therefore seem to be of fundamental importance not only to protect membrane integrity and avoid hemolysis, but also to maintain RBC deformability in response to hydrodynamic forces.

## Materials and methods

### Materials and stock solutions

Unless not indicated otherwise, all chemicals were purchased by Sigma Aldrich (Darmstadt, Germany) and were of the highest purity available. MilliQ quality water was used to prepare all home-made solutions (Millipore, Darmstadt, Germany), Hank's balanced salt solution with Ca^2+^ (HBSS^+^)(1.26 mM CaCl_2_, 0.49 mM MgCl_2_ x 6H_2_O, 0.41 MgSO_4_ x 7H_2_O, 5.33 mM KCl, 0.44 KH_2_PO_4_, 4.12 NaHCO_3_, 137.93 mM NaCl, 0.34 mM Na_2_HPO_4_, 5.56 mM D-glucose) was purchased by Life technologies. Working solutions of *tert*-butylhydroperoxide (*t*-BuOOH) were prepared by diluting 1 M stock in HBSS^+^. Stock solutions of 2-(N,N-diethylamino)-diazenolate-2-oxide (DEA/NO) (50 mM) were prepared in 10 mM NaOH and kept on ice until use. Stock solutions of nitrosated cysteine (CysNO) were prepared as described (Cortese-Krott et al., 2012a). Briefly, 200 mM stock solution of nitrite (VWR, Darmstadt, Germany) and acidified L-cysteine hydrochloride were mixed in equal part, equilibrated to neutral pH, kept on ice in the dark until use and diluted to final concentrations in HBSS^+^.

### Human study and collection of human blood samples

For *ex vivo* analyses, young (20–40 years old), healthy volunteers were recruited and gave written informed consent to participate before enrollment (ClinicalTrials.gov Identifier: NCT02272530). To analyze the effects of hypertension on RBC function, study participants (40–60 years old, average age 50.6 ± 6.9 years for hypertensive subjects and 47.8 ± 5.5 years for healthy subjects) were recruited from the outpatient clinic of the Department of Cardiology, Pneumology and Angiology, University Hospital Düsseldorf. The study was approved by the ethics committee of the Heinrich-Heine-University (HHU) of Düsseldorf, and registered in the coordination center for clinical trials of HHU (KKS, registration ID 201307443). Both studies were conducted in accordance with the Declaration of Helsinki. Clinical protocol and patients' characteristics are described in the Supplemental Information (Table S1).

### Determination of intracellular ROS by flow cytometry

For determination of ROS in RBCs, blood was collected from the antecubital vein of human donors into tubes containing heparin (5000 I.U.), kept on ice and processed within 2 h. Blood was diluted 1:500 with ice cold Dulbecco's phosphate buffered solution (PBS) to reach a final RBC concentration of ~4 × 10^5^ RBC/μL and divided into 1 ml aliquots. Aliquots were treated for 30 min either with 20 μM diclorofluoresceine diacetate (DCF-DA, Invitrogen, Germany) to assess ROS, or with 20 μM Thiol Tracker (TT, Invitrogen) to assess free thiols, or 10 μM 4-amino-5-methylamino-2′,7′-difluorofluorescein-diacetate (DAF-FM, Invitrogen) to assess NO levels, washed, and analyzed in a FACS BD FACSCanto II flow cytometer (BD Bioscience, Heidelberg, Germany) as described (Cortese-Krott et al., 2012a).

### Collection of blood from Nrf2 KO, eNOS KO, and WT mice

All mouse experiments were approved by the LANUV (State Agency for Nature, Environment and Consumer Protection) and conducted in agreement with the German “Tierschutzgesetz” and the “Guide for the Care and Use of Laboratory Animals” of the US National Research Council. The mouse strains used in this study were wildtype C57BL/6J (WT) (Janvier, France), Nrf2 knockout (Nrf2 KO) (BRC No. 01390); kindly provided by Rinken (Koyadai, Tsukuba, Ibaraki, Japan) and endothelial NOS knockout (eNOS KO) (Godecke et al., 1998). Mouse whole blood was drawn in anesthetized mice by cardiac puncture using heparin as anticoagulant.

### Determination of shear-induced elongation of RBCs by ektacytometry

For determination of shear-induced elongation of RBCs, 25 μL of whole blood or RBC pellet (as indicated in the figure legends) were added to 5 mL pre-warmed (37°C) high viscosity PVP solution (RR Mechatronics, Hoorn, The Netherlands) to yield a cell suspension behaving closely to a Newtonian liquid. Using the laser optical rotational red cell analyzer (Lorrca, RR Mechatronics), the elongation index (EI) was measured at a range of different shear stresses (0.30–50 Pa), as indicated in the figures. For *Lineweaver-Burk* transformation, the reciprocal values for the deformability index were plotted against the reciprocal values of the respective shear stress. After linear regression, maximal deformability (EI_max_) and half maximal shear stress (SS_1/2max_) were calculated as described previously (Baskurt and Meiselman, 2013).

### Determination of whole blood bulk viscosity by low shear viscosimetry

Whole blood viscosity measurements were conducted using the LS300 viscometer (proRheo, Althengstett, Germany) as described by Ruef et al. (2014). Briefly 1 mL of treated whole blood suspension was measured at 37°C for a range of different shear rates (0.5–150 1/s).

### RBC isolation

Human and mouse RBC isolation was carried out as described before (Cortese-Krott et al., 2017). Briefly, human whole blood anticoagulated with heparin was transferred into a 20 ml syringe and centrifuged at 800 g for 10 min at 4°C; plasma and buffy coat were aspirated from the top and RBCs were eluted from the bottom of the syringe. The RBC pellet was washed three times with HBSS^+^ by centrifugation at 300 g, for 10 min at 4°C. RBC pellets were kept on ice or equilibrated in pre-warmed HBSS^+^ at 37°C at a cell concentration of 40% hematocrit (hct) on a rolling plate until used for experiments, as indicated below. Mouse RBCs were separated similarly to human RBCs except the centrifugation steps were carried out in Eppendorf tubes.

### Determination of GSH and GSSG in RBCs by liquid chromatography/mass spectrometry

A defined volume (100 μL) of RBC pellet was lysed and protein precipitated in a solution containing 5% sulfosalicylic acid (SSA) and 10 mM N-ethylmaleimide (NEM) in double distilled water (ratio 1:5). After addition of the internal standard (2 mM glutathione ethylester), cell lysis was completed in an ultrasonic bath for 20 s. Afterwards, samples were centrifuged for 10 min, 10,000 g, and at 4°C. Cell pellets were washed once with the same volume buffer used before, centrifuged with the configurations used in the last step, and both supernatants were merged. To separate analytes, a gradient elution on a Zorbax Eclipse Plus C18 RRHD 2.1 × 50 mm 1.8 μm (Agilent) was chosen with 0.1% formic acid in double distilled water (A) and acetonitrile (B) (0–2 min: 99% A, 1% B; 2–7 min: 99% A, B 1% → 1% A, 99% B; 7–12 min: 1% A, 99% B; 12–12.1 min: 1% A, 99% B → 99% A, 1% B, 12.1–16 min 99% A, 1% B) on a 1290 Infinity UPLC system (Agilent, Waldbronn, Germany) and analyzed in an Agilent 6550 iFunnel Accurate-Mass Quadrupole Time-of-Flight Mass Spectrometer (Q-TOF MS). Ionization source was set to positive mode with the configurations: gas temperature 220°C, drying gas 12 l/min, nebulizer 35 psig, sheath gas temperature 330°C, sheath gas flow 11 l/min, Vcapillary 2500 V, nozzle voltage 1000 V, fragmentor 30 V. Data were analyzed using an Agilent MassHunter Workstation Software (Agilent).

### Effects of *t*-BuOOH on GSH/GSSG levels in human RBCs

To analyze the effects of *t*-BuOOH on glutathione (GSH) and glutathione-disulfide (GSSG) levels in human RBCs, washed human RBC suspensions at 40% hct were incubated for 10 min at 37°C with increasing concentrations of *t*-BuOOH (1 nM−10 mM, as indicated in the figure legend), then centrifuged at 300 g for 5 min at 4°C, put on ice, and used immediately for GSH and GSSG determination. We decided to measure reduced and oxidized glutathione since the use of the half redox potential has been criticized as being less relevant in cell physiological processes (Flohe, 2013).

### Effects of *t*-BuOOH and NO donors on RBC deformability and bulk blood viscosity

To analyze the effects of *t*-BuOOH on both shear induced elongation and bulk blood viscosity in parallel, whole blood of each sample was split into 1 mL aliquots, and blood samples were incubated by addition of 100 μL *t*-BuOOH working solutions to reach the concentrations corresponding to 3, 5, and 7 mM *t*-BuOOH. Incubation times from 10 min at 37°C were followed by measurements of deformability (see section Determination of Shear-Induced Elongation of RBCs by Ektacytometry) and viscosity (see section Determination of Whole Blood Bulk Viscosity by Low Shear Viscosimetry) under the same experimental conditions. To examine the effects of *t*-BuOOH on RBCs from Nrf2 KO mice and WT mice, RBC pellets were diluted at 0.8% hct in HBSS^+^, and treated for 20 min with *t*-BuOOH at room temperature (RT). Cells were pelleted by centrifugation (800 g, 4°C, 10 min) and washed with HBSS^+^ and deformability measured as described in section Determination of Shear-Induced Elongation of RBCs by Ektacytometry. To test the effects of NO under conditions used by previous studies (see Table S8), RBC suspensions in HBSS^+^ buffer (see section Determination of Shear-Induced Elongation of RBCs by Ektacytometry) at 1.6% hct were treated with DEA/NO (0–200 μM) and incubated for 10 min at 37°C, pelleted by centrifugation at 800 g for 10 min, and suspended in buffer before analysis. To determine the effects of nitrosothiols, RBC suspensions in HBSS^+^ (see section RBC Isolation) at 25% hct were treated with CysNO as indicated for 10 min at 37°C and pelleted by centrifugation at 800 g for 10 min and re-suspended in HBSS^+^ before analysis. In a further series of experiments, samples were treated with CysNO as indicated for 10 min at 37°C, washed two times with 4 volumes of HBSS^+^ buffer by centrifugation for 5 min at 800 g at 4°C, and then treated with 3 mM *t*-BuOOH for 10 min at 37°C. Cells were pelleted by centrifugation (800 g, 4°C, 10 min) and re-suspended in HBSS^+^ before analysis. In addition, these experiments were carried out in reversal with *t*-BuOOH incubation first and CysNO second.

### Detection of spectrin nitrosation by biotin switch assay

RBC suspensions were prepared by diluting 500 μL of washed RBCs in PBS (137 mM NaCl, 2.7 mM KCl, 10 mM Na_2_HPO_4_, 1.8 mM KH_2_PO_4_, pH = 7.4) containing diethylenetriaminepentaacetic acid (1 μM) and incubated with 10^−9^-10^−2^ M concentrations of CysNO for 30 min at RT. CysNO was subsequently removed by ultrafiltration using Pall Nanosep 10 kDA MWCO spin columns. RBCs were re-suspended in lysis buffer with protease inhibitors [150 mM NH_4_Cl, cOmplete ULTRA Tablets (Roche), in H_2_O]. After protein determination by Lowry (DC Protein Assay, Bio-Rad, München, Germany), a biotin switch assay was carried out to determine the extent of S-nitrosation. The assay was performed according to manufacturer's protocol using a concentration of 0.8 mg/mL protein (*S*-Nitrosylated Protein Detection Kit, Cayman, Ann Arbor, USA). Samples were loaded onto a 7% Nupage Novex Tris/Acetate precast gel, and a Western blot was performed as described elsewhere (Cortese-Krott et al., 2017). Membranes were incubated with a primary mouse anti-spectrin antibody (1:1000) from Sigma Aldrich at 4°C overnight, followed by parallel assessment of spectrin and biotin signals on the same nitrocellulose membrane using anti-mouse Cy3-coupled antibodies (Thermo Fisher) and strepdavidin- coupled to the Cy5 fluorophore (Thermo-Fisher).

### Statistics

All data were analyzed by using GraphPad Prism PC software (version 6.01; Graph Pad, La Jolla, CA, USA), and are expressed as means ± S.E.M. of n individual samples as stated in Results and Figure legends. Statistical comparisons between groups were performed by one-way or two-way ANOVA as required by experimental setting, followed by an appropriate multiple comparison *post-hoc* test (Dunnet's or Sidak's) or *t*-test as indicated in the figure legend. Comparison of data from hypertensive subjects with aged-matched controls was carried out by non-parametric Mann-Whitney-*U*-testing. *P* < 0.05 was considered statistically significant.

## Results

### RBCs from hypertensive patients have decreased deformability and increased intracellular ROS levels

The bulk of data on the effects of pathological conditions on RBC deformability show that RBCs of patients with hypertension have significantly decreased RBC deformability (Sandhagen et al., 1990; Vaya et al., 1992; Cicco and Pirrelli, 1999; Cicco et al., 1999a,b, 2001; Turchetti et al., 1999; Vetrugno et al., 2004; Radosinska and Vrbjar, 2016). Hypertension is a complex condition, which is accompanied by endothelial dysfunction and reduced NO bioavailability. A characterization of RBC deformability in relation to redox status/NO levels in RBCs of hypertensive patients was never carried out before. Therefore, we carried out an observational study where RBCs from hypertensive patients were compared with RBCs from aged matched individuals without hypertension (see supplemental information Table S1 for patient characteristics). We measured RBC characteristics including RBC deformability, ROS levels, NO levels, and total free thiol levels.

**Figure 1:**
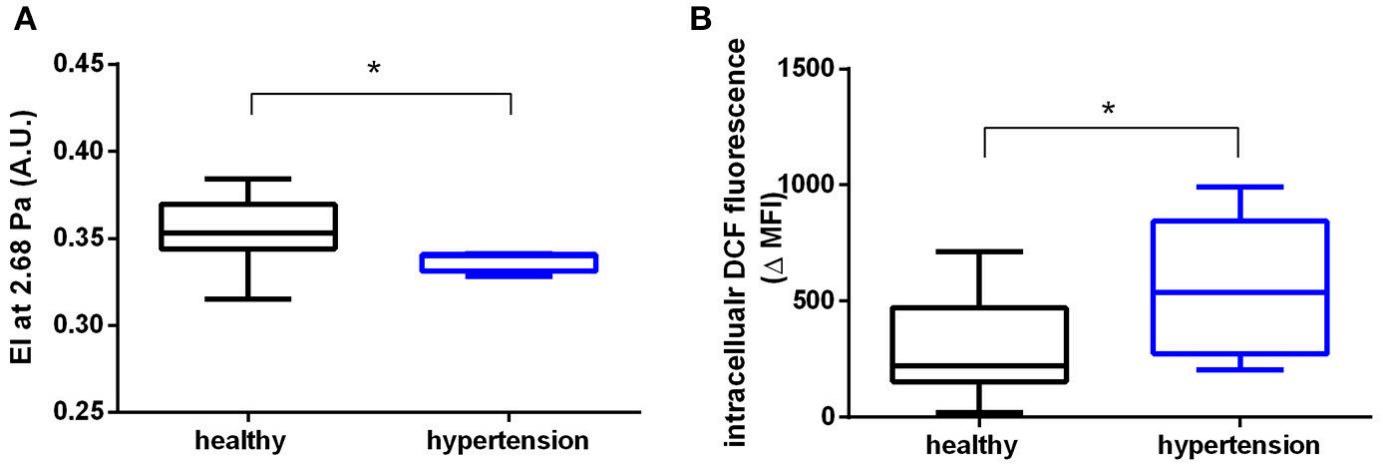
RBCs from patients with hypertension display decreased RBC deformability and increased ROS levels. **(A)** Decreased elongation index (EI) measured in RBCs of hypertensive (*n* = 4) and healthy (*n* = 9) participants at a shear stress of 2.68 Pa. ^*^Mann-Whitney-*U* test, *p* < 0.05. **(B)** Increased levels of ROS in RBCs of hypertensive participants (*n* = 9) measured as intracellular dichlorofluorescein (DCF) fluorescence values and compared to healthy controls (*n* = 11) (MFI, median fluorescence intensity; ΔMFI = MFI of loaded RBCs—MFI unloaded RBCs) ^*^Mann-Whitney-*U* test, *p* < 0.05. All ektacytometric measurements were carried out from whole blood diluted in PVP solution and measured in a range of shear stresses of 0.3–10 Pa.

We found that RBCs from patients with hypertension showed significantly decreased shear-induced elongation, as assessed by ektacytometry in a LORCA; in fact, we found that the EI was decreased at low shear rates (1.73 Pa: healthy 0.275 ± 0.022, hypertension: 0.2568 ± 0.007; *p* = 0.056; Table S2) and at 2.68 Pa (Figure 1A), while no difference was detectable at higher shear rates. The data were analyzed using the Lineweaver-Burk transformation (Baskurt and Meiselman, 2013) by plotting the reciprocal values of EI against the reciprocal shear stress ($r_{\text{hypertension}}^{2}$ = 0.91, $r_{\text{healthy}}^{2}$ = 0.83). We found that SS_1/2max_ and EI_max_ were not significantly different in hypertensive subjects as compared to age-matched controls (Table S2).

We also found a significant increase of intracellular levels of ROS (assessed by intracellular DCF fluorescence by flow cytometry) in RBCs from hypertensive patients as compared to age-matched controls (Figure 1B). However, RBCs from patients with hypertension showed no changes in total (high molecular weight and low molecular weight) “free”/reduced thiol levels in RBCs; these were analyzed by intracellular fluorescence of a Thiol Tracker® (TT), which reacts with “free”/reduced intracellular sulfhydryl (-SH) groups (Table S1). Likewise, the levels of NO metabolites in RBCs measured by intracellular DAF fluorescence (Cortese-Krott et al., 2012a,b) as well as intracellular nitrite concentrations were similar in both study groups (Table S1). Taken together, these data demonstrate that RBCs from hypertensive patients have decreased EI at low shear stresses and increased intracellular ROS, while intracellular NO levels and total free thiol levels were preserved.

### Oxidative stress induced by treatment with *tert*-butylhydroperoxide decreases redox reserve (total GSH) and impairs RBC deformability and whole blood viscosity

**Figure 2:**
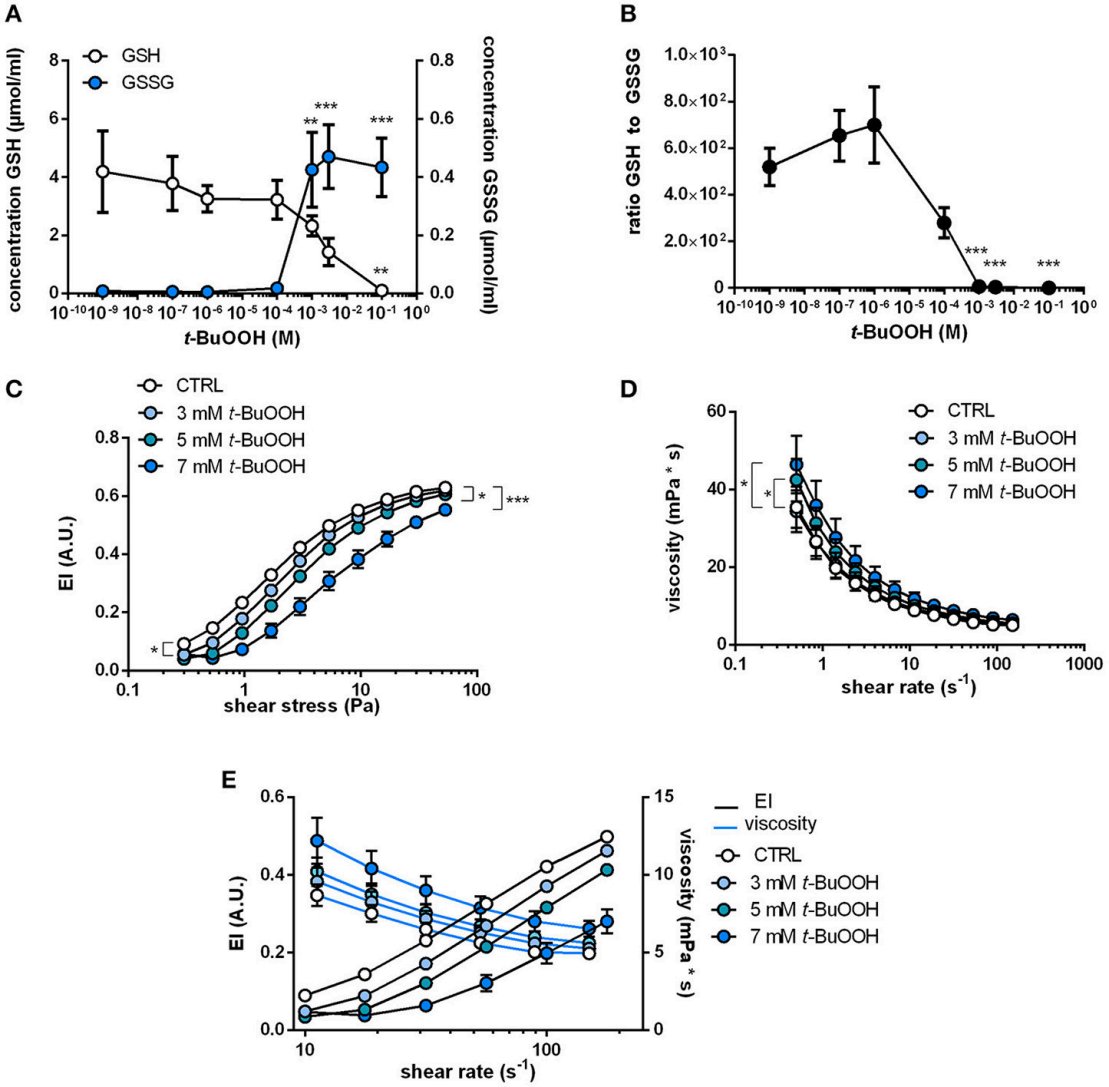
Changes in intracellular redox state by treatment with *t*-BuOOH decrease RBC deformability and increase blood viscosity in a concentration-dependent fashion. **(A)** Changes in intracellular GSH and GSSG levels upon treatment of RBCs with different concentrations of *t*-BuOOH (0 M, 10^−9^ M, 10^−7^ M, 10^−6^ M, 10^−4^ M, 10^−3^ M, 3 × 10^−3^ M, 10^−1^ M). Free/reduced GSH levels decreased with a simultaneous increase of GSSG, the oxidized form of glutathione (*n* = 4). GSH: one-way RM ANOVA *p* = 0.0218, ^**^p < 0.01, Dunett's test vs. untreated control. GSSG: one-way RM ANOVA p < 0.0001, ^**^p < 0.01, ^***^ *p* < 0.001, Dunett's test vs. untreated control. **(B)** Ratio of free/reduced GSH and GSSG calculated from the values in **(A)**. One-way RM ANOVA *p* < 0.0001, ^***^ *p* < 0.001, Dunett's test vs. untreated control. **(C)** Relationship between elongation index (EI) and applied shear stress (0.30–53.33 Pa) of whole blood samples treated with increasing concentrations of *t*-BuOOH (3–7 mM) showing that *t*-BuOOH impairs RBC deformability in a concentration-dependent fashion (*n* = 6). Two-way RM ANOVA *p* < 0.001 and Dunett's test vs. untreated control; ^*^p ≤ 0.05, ^***^p < 0.001 (statistically significant for 3 mM from shear stresses between 0.3 and 9.48 Pa; for 5 and 7 mM over the whole range of shear stresses). **(D)** Analysis of blood viscosity of the same samples described in **(C)** over a range of different shear rates (*n* = 6). Two-way ANOVA *p* = 0.0004 and Dunnett's test vs. untreated control, ^*^ *p* ≤ 0.05 (for 5 mM statistically significant from shear rates between 0.5 and 3.98 s^−1^ and for 7 mM from shear rates between 0.5 and 11.22 s^−1^). **(E)** Comparison of changes in viscosity and EI at matching shear rates show inverse relationship between the two parameters.

Oxidation of cytoskeletal proteins in RBCs were shown to induce changes in RBC shape and membrane elasticity. To verify that ROS cause changes in RBC deformability, we tested the effect of a prototypical pharmacological oxidative challenge *t*-BuOOH on intracellular redox state and RBC deformability. This treatment was found to induce oxidation of the cytoskeletal protein spectrin (Lii and Hung, 1997) and hemoglobin (Gorbunov et al., 1997). To analyze intracellular redox changes in RBCs following treatment with *t*-BuOOH, we measured intracellular concentrations of reduced glutathione (“free GSH”) and oxidized glutathione (GSSG) and calculated the concentration of total GSH (GSH + 2 ^*^ GSSG; indicative of the antioxidant reserve) and the molar ratio (GSH/GSSG), which is indicative of the redox state of the cell. Treatment with concentrations of *t*-BuOOH higher than 3 mM decreased free intracellular GSH levels in a concentration-dependent fashion (starting at levels >100 μM), while GSSG levels simultaneously increased (Figure 2A). The ratios of GSH and GSSG were stable at low concentrations of *t*-BuOOH, but began to decrease and started to drop with a *t-*BuOOH concentration of 10^−6^ M (Figure 2B), as a result of depletion of reduced GSH and increase of GSSG at high *t*-BuOOH concentrations. This is in accordance with previous studies showing that high non-physiological concentrations of *t*-BuOOH are needed to induce oxidative stress in RBCs (Trotta et al., 1983; Rice-Evans et al., 1985; Lii and Hung, 1997).

To evaluate whether those redox changes induced by *t*-BuOOH influence RBC deformability, we treated blood samples with increasing concentrations of *t*-BuOOH and measured both shear-induced elongation (by using a LORCA) as well as “bulk” blood viscosity (by using a low shear viscosimeter). *t-*BuOOH in concentrations of < 3 mM did not affect any of the parameters of RBC deformability while significantly decreasing EI at discrete shear stresses at concentrations ≥ 3 mM (Figure 2C). However, EI_max_ and SS_1/2max_ (calculated according to Lineweaver-Burk transformation) were not significantly different from control. It is important to point out that in these experiments at high concentrations of *t*-BuOOH the linearity of the Lineweaver-Burk relationship was lost (Table S3), indicating that this transformation method is not applicable in the described experimental conditions.

Assessments of bulk blood viscosity by low shear viscosimetry revealed that *t-*BuOOH in concentrations of 5 mM and 7 mM significantly increased blood viscosity (Figure 2D). By comparing changes in EI and whole blood viscosity assessed at the same shear rates we found that *t-*BuOOH-induced decrease of deformability corresponded to a simultaneous increase in bulk viscosity, and that both parameters were dependent on the concentration of *t*-BuOOH (Figure 2E). We can exclude membrane damage as we did not detect any increases of hemolysis induced by all applied treatments (Table S3).

Taken together, these data indicate that *t*-BuOOH induces changes in shear-induced elongation of RBCs and affects the overall rheological properties of RBCs in whole blood. Interestingly, those events appear to occur only at *t*-BuOOH concentrations that fully consumed the antioxidant capacity (total GSH) and redox reserve (GSH/GSSG ratio) in RBCs.

### Effects of *t*-BuOOH on RBC deformability in Nrf2 KO mice

**Figure 3:**
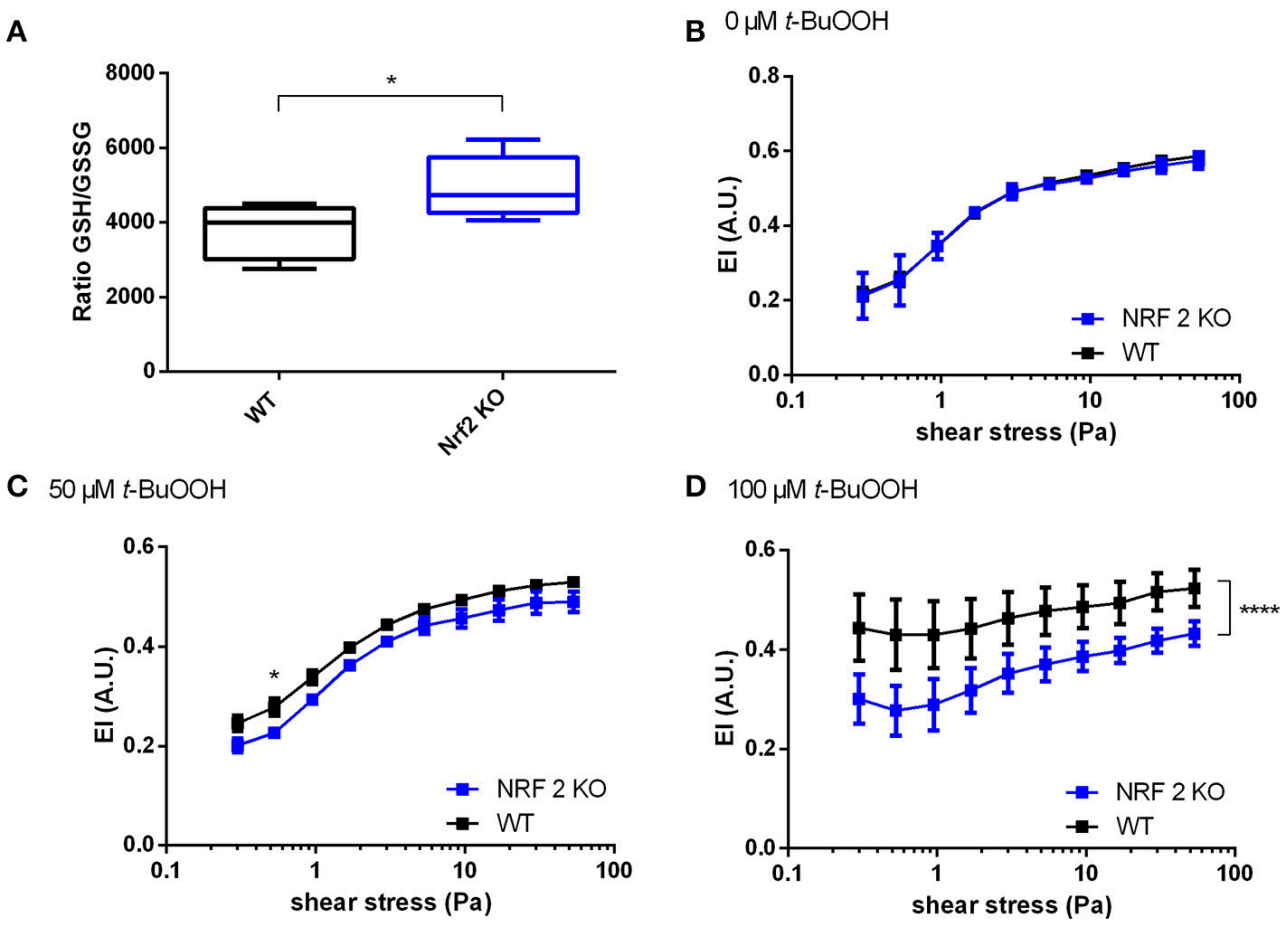
Increased susceptibility of RBCs from Nrf2 KO mice to *t*-BuOOH-induced impairment of deformability. **(A)** The ratio of GSH/GSSG in Nrf2 KO is increased as compared to WT mice (*n* = 5),^*^p < 0.05 student's *t*-test. **(B)** No difference exists between RBC deformability curves in wild type (WT) mice and Nrf2 KO mice as determined by ektacytometric analysis of RBC pellet two-way RM ANOVA *p* = 0.4497. **(C)** RBCs from Nrf2 KO mice show a significant decrease of EI values after treatment with 50 μM *t*-BuOOH to WT controls (*n* = 6). Two-way ANOVA *p* = 0.0341 and Sidak's test; ^*^p ≤ 0.05, **(D)** RBCs from Nrf2 KO mice show a significant decrease of EI values after treatment with 100 μM *t*-BuOOH as compared to WT controls (*n* = 6). Two-way ANOVA *p* = 0.0095 and Sidak's test; ^****^p < 0.0001 over the whole range of shear stresses. All ektacytometric measurements were carried out from washed RBC diluted in PVP and measured in a range of shear stresses of 0.3–53.33 Pa.

Next we analyzed the effects of *t*-BuOOH on RBCs from Nrf2 KO mice. This mouse strain was chosen, because lack of Nrf2 induces deficiency of a number of antioxidant/reducing enzymes (Suzuki and Yamamoto, 2015) and may serve as a model for increased susceptibility to oxidative damage. This strain showed increased susceptibility to oxidative challenges (Rangasamy et al., 2005), as well as decreased levels of total GSH in heart and aortic tissue (Erkens et al., 2015). However, the impact of oxidative stress on RBC deformability has been never investigated so far. Unexpectedly, RBCs from Nrf2 KO mice did not show any significant differences in redox reserve (total intracellular GSH); instead we found a compensatory decrease in oxidized GSH (GSSG), which in the absence of any changes in free GSH levels translates into an improved 1.3-fold GSH/GSSG ratio (redox reserve) as shown in Figure 3A and Table 1. Accordingly to our hypothesis that redox state is one of the major determinants of RBC deformability, there were no differences in deformability of RBCs at baseline (Figure 3B) between Nrf2 KO and WT mice. However, when RBCs were challenged with high concetrations of *t*-BuOOH, clear differences in RBC deformability became apparent. Specifically, treatment of RBCs from Nrf2 KO mice with 50 or 100 μM *t-*BuOOH induced a significant impairment of deformability of RBCs from Nrf2 KO mice at both low and high range of shear stresses (Figures 3C,D) and significantly decreased EI_max_ as compared to WT (Table S4). These findings suggest that the compensatory increase in the GSH/GSSG ratio of RBCs from Nrf2 KO seen under resting conditions is not enough to protect RBCs from oxidant-induced impairment of RBC deformability, and are indicative of impaired resilience against oxidants of Nrf2 KO RBCs as compared to WT mice.

**Table 1:** GSH content and redox state in RBCs from eNOS KO, Nrf2 KO, and WT mice.

	**RBC eNOS KO**	**RBC Nrf2 KO**	**RBC WT**
GSH (nM)	3.76^*^10^6^ ± 0.26^*^10^6^	3.77^*^10^6^ ± 0.14^*^10^6^	3.93^*^10^6^ ± 0.23^*^10^6^
GSSG (nM)	946.0 ± 161.3	781.5 ± 68.6	1057.7 ± 46.4
Total GSH (nM)	3.76^*^10^6^ ± 0.26^*^10^6^	3.77^*^10^6^ ± 0.14^*^10^6^	3.94^*^10^6^ ± 0.23^*^10^6^
GSH/GSSG	3974.6	4821.5	3718.3

*Intracellular reduced/”free” GSH, oxidized GSH (GSH disulfide), total GSH (calculated by GSH and GSH disulfide levels), and ratio of GSH to GSH disulfide measured in RBCs by mass spectroscopy*.

### RBC deformability is not affected by endogenously produced or exogenously applied NO

Another determinant of RBC deformability described in literature is both exogenously applied or endogenously produced NO by red cell eNOS. Both endogenously and exogenously applied NO have been described to affect RBC deformability *ex vivo*, assessed either as RBC filterability or as shear-induced deformation by ektacytometry (Korbut and Gryglewski, 1993; Bor-Kucukatay et al., 2003; Kleinbongard et al., 2006; Grau et al., 2013). These data stand in contrast to more recent data showing no effects of sodium nitroprusside (Barodka et al., 2014b), or NO donors or NOS inhibitors on RBC deformability determined by ektacytometry (Belanger et al., 2015), calling for a careful reassessment of those earlier findings.

**Figure 4:**
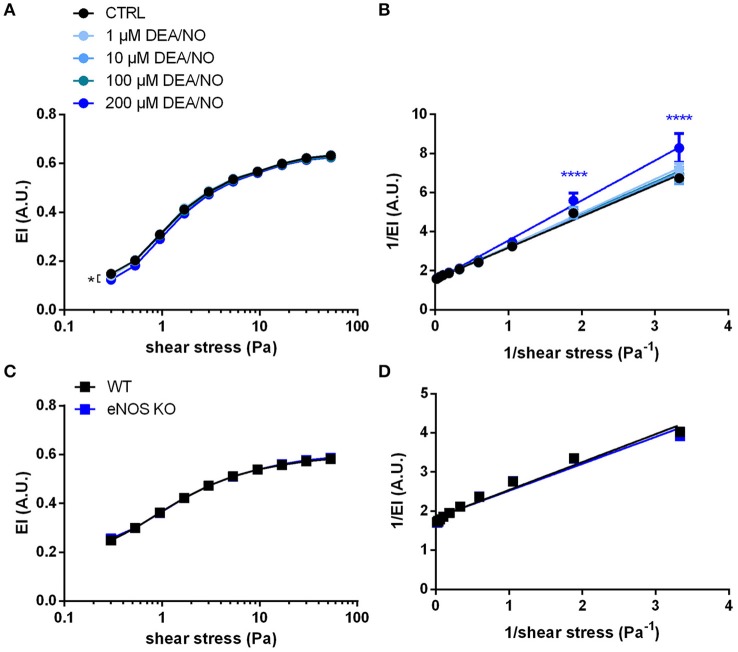
NO does not influence RBC deformability measured by ektacytometry: **(A)** Treatment with the NO donor DEA/NO did not affect EI; the highest concentration (200 μM DEA/NO) significantly decreased RBC deformability. Two-way RM ANOVA *p* < 0.0001 and Dunnett's test vs. untreated control, ^*^p ≤ 0.05 (statistically significant for 200 μM DEA/NO in the range of shear stresses between 0.3–5.3 and 30–53.33 Pa). **(B)** The Lineweaver–Burk transformation resulted in linear regression curves that are not significantly different from each other (*n* = 6). Two-way ANOVA *p* = 0.0015 and Dunnett's test vs. untreated control, ^****^p < 0.0001. **(C)** Deformability curves of RBCs from eNOS KO mice do not differ from that of WT mice. Two-way ANOVA *p* = 0.4725. **(D)** Lineweaver-Burk transformation of curves in C do not show any significant difference (*n* = 6). Two-way ANOVA *p* = 0.6029 All ektacytometric measurements were carried out from washed RBCs diluted in PVP solution and measured in a range of shear stresses of 0.3–53.33 Pa.

Therefore, to analyze the effects of NO on RBC deformability in our experimental setting, RBCs were exposed to increasing concentrations of the NO donor DEA/NO. DEA/NO was chosen because it is well known to spontaneously release NO in a controlled and predictable manner (differently from sodium nitroprusside, which needs to be metabolized in cells and releases also ${\text{O}}_{2}^{-}$) (Feelisch, 1998). In our hands, the incubation of RBCs with the spontaneous NO donor DEA/NO (1–100 μM) did not significantly influence deformability indices over the whole range of shear stresses analyzed (Figure 4A). Lineweaver-Burk transformations of this data (Figure 4B) followed by calculation of EI_max_ and SS_1/2max_ (Table S5) resulted in values not significantly different from control conditions at concentrations ranging from 1 to 100 μM. Instead, treatment with the highest concentration of DEA/NO (200 μM) decreased RBC deformability significantly (Figure 4A). These data indicate that exposure of RBCs to exogenous NO does not influence RBC deformability in a beneficial manner.

It is well-established that RBCs carry a functional eNOS (Kleinbongard et al., 2006; Cortese-Krott et al., 2012b) and, according to published data by Bor-Kucukatay *et al*. RBC eNOS-derived NO is able to improve RBC deformability (Bor-Kucukatay et al., 2003). To examine the effects of endogenously produced NO in RBCs, we compared RBC deformability of global eNOS KO mice with RBC deformability of WT mice and found that genetic deficiency for eNOS had no effect on RBC deformability. This was evidenced by essentially superimposable deformability curves measured at discrete shear stresses in WT and eNOS KO (Figure 4C) and virtually identical calculated values for EI_max_ and SS_1/2max_ after Lineweaver-Burk transformation (Figure 4D, Table S6). Likewise, the levels of GSH and GSSG or their ratios were not different between eNOS KO and WT strains (Table 1). Therefore, RBC deformability is neither affected by treatment with NO donors nor by endogenous NO formation/synthesis by red cell eNOS.

### Treatment with *S*-nitrosothiols leads to nitrosation of cytoskeletal proteins and rescues from *t*-BuOOH-mediated damage

**Figure 5:**
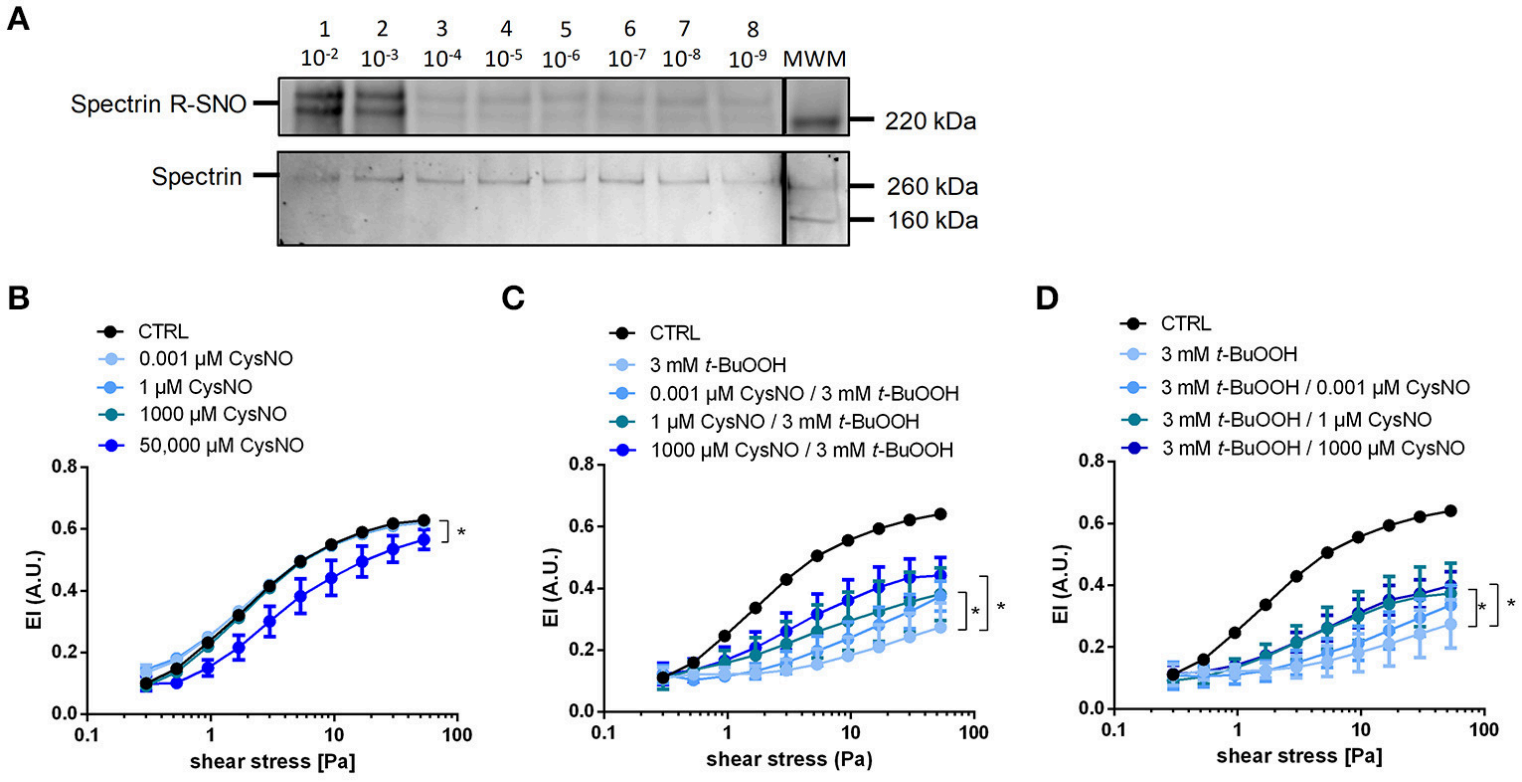
Treatment with CysNO leads to *S*-nitrosation of spectrin and protects RBCs from *t*-BuOOH-induced impairment of RBC deformability. **(A)** *S*-nitrosation of spectrin following treatment of RBC suspension with 10^−9^ to 10^−2^ M CysNO for 30 min as assessed by biotin switch assay (upper lane). Total spectrin, on the same membrane determined by Western blot. Each lane is labeled with the treatment concentration of CysNO. Lane MWM is the corresponding molecular weight marker (for whole images of whole membrane please refer to Figure S4). **(B)** Increasing concentrations of CysNO did not affect RBC deformability, except at the highest concentration (50,000 μM CysNO). Two-way RM ANOVA *p* = 0.0059 and Dunnett's test vs. untreated control, ^*^p ≤ 0.05 (for 50,000 μM statistically significant over a range of shear stresses from 0.53 to 53.33 Pa). **(C)** Pre-incubation of RBCs with different concentrations of nitrosating agent CysNO significantly rescued RBC deformability after treatment with 3 mM *t-*BuOOH (*n* = 5). Two-way RM ANOVA *p* < 0.0003 and Tukey's test; ^*^p ≤ 0.05 vs. 3 mM *t*-BuOOH (statistically significant for 1 μM CysNO pre-incubation in the shear stresses from 5.33 to 53.33 Pa and for 1000 μM CysNO pre-incubation in the shear stress range from 3 to 53.33 Pa). **(D)** Post-incubation of RBCs with different concentrations of nitrosating agent CysNO significantly rescued RBC deformability after treatment with 3 mM *t-*BuOOH (*n* = 5). Two-way RM ANOVA *p* < 0.0003 and Tukey's test; ^*^p ≤ 0.05 vs. 3 mM *t*-BuOOH (statistically significant for 1 μM CysNO and 1000 μM CysNO in the range of shear stresses from 5.33 to 53.33 Pa). Deformability index of RBC pellets measured in a range of shear stresses of 0.3–53.33 Pa.

There is evidence for a role of nitrosation of critical thiols in the cytoskeletal protein spectrin in the regulation of RBC deformability (Grau et al., 2013). To study whether *S*-nitrosation reactions impact RBC deformability, we incubated RBCs with the nitrosothiol CysNO. We found that 1 mM CysNO indeed increases *S-*nitrosation of the cytoskeletal protein spectrin, as determined by biotin switch assay and Western blotting (Figure 5A; Figure S4). However, similar to what we observed with the NO donor DEA/NO, the treatment of RBCs with CysNO at concentrations ranging from 0.001 to 1000 μM did neither affect EI, EI_max_, and SS_1/2max_, nor bulk viscosity beneficially in CysNO-treated RBCs compared to untreated cells (Figure 5B, Figure S2, Table S7). Similar to what we observed for 200 μM DEA/NO, also high concentrations of CysNO (50,000 μM) decreased RBC deformability significantly (Figure 5B).

To analyze the effects of CysNO on *t*-BuOOH-induced impairment of RBCs deformability, we pre-incubated RBCs with CysNO, washed them, and afterwards treated them with *t*-BuOOH. The deformability curves showed that pre-treatment with CysNO at concentrations between 1 and 1000 μM significantly improved RBC deformability (Figure 5C). These effects were especially profound with high shear stresses as demonstrated by increase in EI (Figure 5C), while EI_max_ and SS_1/2max_ were not significantly different (Table S7). Interestingly, treatment with CysNO after *t*-BuOOH incubation resulted in comparable protection of RBC deformability (Figure 5D).

Taken together, treatment of RBCs with nitrosothiols led to *S*-nitrosation of the cytoskeletal protein spectrin but did not affect RBC deformability *per se*, as assessed by measurements of shear-dependent elongation and bulk viscosity. However, nitrosothiols protected RBCs against *t*-BuOOH-mediated impairment of RBC deformability.

## Discussion

Studying the molecular mechanisms controlling RBC deformability is of fundamental importance to understand physiology and pathophysiology of RBCs. Oxidative modifications of the RBC cytoskeleton were shown to decrease deformability (Sinha et al., 2015). In addition, NO was proposed to participate in the regulation of RBC deformability (Bor-Kucukatay et al., 2003; Grau et al., 2013), but these findings were not reproduced by other laboratories (Barodka et al., 2014b; Belanger et al., 2015; Cortese-Krott et al., 2017).

In this work, we investigated if and how changes in intracellular redox status, levels of ROS, NO metabolites, and nitrosation reactions may affect RBC deformability. We found that: (1) in hypertensive patients decreased RBC deformability was accompanied by an increase in intracellular ROS levels, with unchanged intracellular NO levels and total free thiols; (2) treatment with high concentrations of *t*-BuOOH provoked changes of intracellular redox state, induced changes in RBC deformability and increased blood viscosity; (3) effects of high concentrations of *t*-BuOOH on RBC deformability were more pronounced in RBCs from mice lacking the Nrf2-dependent antioxidant response; (4) neither NO administration by NO donors nor nitrosothiols nor the lack of eNOS in eNOS KO mice induced changes in RBC deformability; (5) treatment with a nitrosothiol rescued RBCs from adverse changes induced by high concentrations of *t*-BuOOH.

Taken together, these findings suggest that NO itself does not affect RBC deformability *per se*, but preserves RBC deformability in conditions of oxidative stress. The mechanisms linking NO-mediated biochemical pathways and modifications regulating RBC deformability induced by ROS need to be identified in future studies.

### Relationship between intracellular redox state and mechanical properties of RBCs

The deformability of RBCs is defined as the ability of RBCs to change their shape in response to external hydrodynamic forces, which are exerted on the cells. Intrinsic determinants of RBC deformability are (1.) the geometry of the cells (i.e., the relationship between surface area and cellular volume), (2.) intracellular viscosity, and (3.) the elastic properties of the membrane/membrane viscosity (Chien, 1987; Mohandas and Gallagher, 2008). These three parameters can be seen as species-unspecific, which enabled us to compare human and murine RBCs. RBC deformability measurements have been carried out by analyzing stress-strain relationships; i.e., by applying a deforming force (on the whole cell or on a small portion of the membrane) and analyzing the resulting cell deformation by direct microscopic observation or by indirect estimations, such as analyzing changes in light diffraction pattern during ellipsoidal deformation (ektacytometry) or changes in bulk viscosity.

Under these conditions, the overall deformation of RBCs depends on both intrinsic properties of the RBC (geometry, intracellular viscosity, membrane viscosity) and on extracellular bulk viscosity; in turn the latter depends on a variety of factors, including cell concentration, plasma/medium viscosity, hydrodynamic forces, cell aggregation, and cell-cell interactions (Chien, 1987). Thus, depending on the forces applied and the experimental setup used for the assessment, each system induces changes in RBC shape that are more or less influenced by changes in cell geometry, intracellular viscosity, or extracellular medium composition in a different way.

This is particularly relevant if the biological chemistry and biochemistry of changes in RBC cell deformability by oxidants or NO metabolites are investigated in a system as complex as the RBCs (and may help to explain some of the discrepancies found in the literature regarding the role of NO and oxidant in RBC deformability, as discussed in detail below in paragraph section Protective Effects of Treatment With Nitrosothiols on *t*-BuOOH-Induced Decrease of RBC Deformability).

In our study, RBC deformability was measured, first as changes in cell elongation in response to shear stress in diluted cell suspensions in a highly viscous PVP medium (by LORCA), and second as changes in bulk viscosity of RBC suspensions at physiological hct in response to a range of shear rates. In the LORCA measurements, the deforming force (τ) is dependent on the two components shear rate (γ) and medium viscosity (η_o_) and is defined as “bulk shear stress” and expressed in Pa.

$$\begin{array}{r} \tau =\gamma \times \eta_{\text{o}}. \end{array}$$

In the LORCA the highly viscous, Newtonian medium is the PVP solution (η_o_), and therefore the degree of RBC deformation is mainly determined by the intrinsic viscosity parameters of the RBC, i.e., intracellular viscosity (η_i_) and membrane viscosity (η_m_); parameters such as cell geometry, or cell adhesion play a minor or non-existent role in ektacytometric measurements (Chien, 1987).

In the LAS300 low-shear viscosimeter measurements are carried out using whole blood; here the blood itself behaves like a highly concentrated cell suspension analogous to a highly viscous artificial medium, with the difference of being a non-Newtonian fluid. Therefore, in low-shear viscosimetry shear induced deformation depends on both intrinsic and extrinsic erythrocyte properties, including intracellular viscosity (η_i_) and membrane viscosity (η_m_) (like in ektacytometry), but also cell volume, cell-cell interactions and blood plasma viscosity (Musielak, 2009).

Intracellular viscosity (η_i_) is mainly dependent on the concentration and physicochemical properties of hemoglobin (Mohandas et al., 1980; Chen and Kaul, 1994), while membrane viscosity η_m_ depends on the elasticity of the spectrin cytoskeleton and its interactions with transmembrane proteins and/or hemoglobin. Therefore, according to these measurement principles described above, changes in RBC deformability assessed by LORCA (or similar methods measuring stress-induced elongation) as a consequence of oxidative stress are likely resulting from changes of the structure/integrity and concentration of hemoglobin and/or of cytoskeletal proteins like spectrin (Corry et al., 1980; Chen and Kaul, 1994; Kiefer and Snyder, 2000; Mandal et al., 2002).

To induce oxidative stress in RBCs, RBCs were treated with high non-physiological concentrations of *t*-BuOOH. The conditions chosen for the experiments were based on previous data/findings showing that millimolar concentrations of *t*-BuOOH are needed to induce changes on RBC deformability (Corry et al., 1980; Trotta et al., 1983). Based on this published observations, we carried out experiments to carefully characterize the effects of increasing concentrations of *t*-BuOOH ranging from 10^−9^ to10^−1^ M on intracellular redox status (assessed as GSH/GSSG ratio), as well as RBC membrane integrity and hemolysis. Here, we found that only the concentrations of *t*-BuOOH, which decreased the intracellular GSH/GSSG ratio, significantly affected shear-induced elongation and bulk blood viscosity (Figure 2). Our findings are in agreement with previous results demonstrating that only high millimolar concentrations of *t*-BuOOH induce hemoglobin oxidation and changes in viscosity of hemoglobin solutions, membrane lipid peroxidation (Trotta et al., 1983), as well as oxidative modifications of cytoskeletal proteins (including crosslinking). Interestingly, oxidants are also known to promote crosslinking of hemoglobin to cytoskeletal proteins (Nagababu et al., 2010), probably further contributing to an impairment of RBC deformability.

This protection may be afforded by both exceptionally high concentrations of intracellular antioxidant systems and a highly efficient reducing capacity that allows rapid recycling of oxidized low-molecular weight constituents, such as glutathione and/or ascorbate (Kuhn et al., 2017). In fact, as also shown here by analyzing RBCs from Nrf2 KO mice, the antioxidant capacity in RBCs is linked to the susceptibility to oxidative damage. In Nrf2 KO mice, the levels of antioxidant and detoxifying enzymes are significantly decreased (Suzuki and Yamamoto, 2015), and Nrf2 KO mice are more sensitive to oxidative damage. Therefore, we expected Nrf2 KO mice to be more susceptible to oxidative stress of high concetrations of *t*-BuOOH.

In fact, we found that RBC of Nrf2 KO mice showed an increased susceptibility to *t*-BuOOH-induced impairment of RBC deformability. Specifically, the effects of *t*-BuOOH on the elongation curves of Nrf2 KO RBCs were significant already at lower concentrations of the peroxide as compared to WT RBCs. In contrast, in untreated cells (at baseline) RBCs from Nrf2 KO mice showed fully preserved antioxidant reserve (total GSH levels), and increased GSH/GSSG ratio in RBCs. This is in contrast to what we and others observed in other organs (aorta, heart) of these mice, where a decrease in total GSH and expression of GSH synthesizing enzymes was observed (Enomoto et al., 2001; Erkens et al., 2015). Therefore, although RBCs of Nrf2 KO mice are able to compensate for genetic deficiency of antioxidant enzymes by increase of total GSH in the RBCs, they are more susceptible to *t*-BuOOH-induced impairment of RBC deformability.

In the present study we were specifically interested in seeing how oxidative stress resulting from an acute challenge with of an oxidant leads to a drop in GSH/GSSG ratio and affects RBC deformability. As pointed out before, the concentrations of *t*-BuOOH needed to induce such changes were in the millimolar range, and therefore 10 to 1,000-folds higher as compared to physiological peroxide concentrations. Experimental setups analyzing the effects treatments with lower concentrations of peroxide may provide further information on how RBCs cope with oxidative stress induced by physiological concentrations of oxidants produced by vascular or blood cells, or due to the presence of xenobiotics in the circulation. Although the concentrations of *t*-BuOOH applied were very high, we did not observe significant membrane damage and hemolysis in our samples (Figure S3). According to previously published data (Trotta et al., 1983), even higher concentrations of peroxide (H_2_O_2_ or *t*-BuOOH) were required to induce hemolysis, further emphasizing the exceptional high antioxidant reserve capacity of RBCs.

This peculiarly high redox buffering capacity of RBCs is likely due to the need of keeping hemoglobin (Hb) in a reduced-Fe^2+^, oxygen-binding form to function as efficient O_2_ delivery system. Interestingly, the major source of ROS in RBCs is thought to be Hb itself through the autoxidation reaction of Hb (Kuhn et al., 2017). Moreover, redox dysregulation, due to genetic deficiency of enzymes providing redox equivalents (such as GAPDH) or antioxidant/detoxifying enzymes (such as glutathione peroxidase) is associated with increased membrane fragility and intravascular hemolysis, and hemolytic anemia (Kuhn et al., 2017).

Taken together, these results demonstrate that changes in the intracellular redox reserve (total GSH) of RBCs may profoundly affect their intrinsic RBC flexibility prior to the loss of membrane integrity and hemolysis.

### Protective effects of treatment with nitrosothiols on *t*-BuOOH-induced decrease of RBC deformability

There is a large amount of literature describing the effects of oxidants, alkylating agents, Ca^2+^/Ca^2+^ ionophore, NO donors and nitrosothiols, as well as NOS inhibitors, and the sGC inhibitor ODQ on RBC deformability (Fischer et al., 1978; Corry et al., 1980; Mesquita et al., 2001; Bor-Kucukatay et al., 2003; Barodka et al., 2014b). However, especially in the case of NO donors and inhibitors of the eNOS/sGC pathway, some of these effects could not be reproduced by other laboratories including ours (Barodka et al., 2014b; Belanger et al., 2015; Cortese-Krott et al., 2017).

The effects of NO on RBC deformability were attributed to the ability of NO donors (Bor-Kucukatay et al., 2003; Riccio et al., 2015) or eNOS-derived NO (Grau et al., 2013) to induce nitrosation reactions of intracellular proteins, including hemoglobin (Riccio et al., 2015) and the cytoskeletal protein spectrin (Grau et al., 2013). We found that neither treatment with NO donors nor nitrosothiols affected RBC deformability and blood viscosity under the conditions applied in this study.

These data are in striking contrast to previously published results (Bor-Kucukatay et al., 2000, 2003; Grau et al., 2013), but are supported by recent findings of two independent groups showing no effects of different NO donors on deformability of human RBCs (Barodka et al., 2014b; Belanger et al., 2015) as assessed by a microfluidic ektacytometric technique (Barodka et al., 2014b), or by osmotic gradient ektacytometry (Belanger et al., 2015). Accordingly, using RBCs from eNOS KO mice, we could not confirm the role of red cell eNOS in regulating RBC deformability (assessed by ektacytometry). Accordingly, treatment with the NOS inhibitor ETU *in vitro* or *in vivo* did not affect RBC deformability in WT (Figure S1).

We were unable to identify any methodological difference in the ektacytometric measurements that might explain these discrepancies (please refer to Table S8 for a comparison of the experimental setups). In previous publications, it was shown that NOS inhibition changed human RBC filterability (Bor-Kucukatay et al., 2000; Kleinbongard et al., 2006), which is dependent on both, RBC deformability and aggregability (Chien, 1987). In chicken, we have found that treatment of RBCs with a NOS inhibitor decreased RBC deformability and RBC velocity in the microcirculatory system of the chorioallantoic membrane (Horn et al., 2011). However, chicken RBCs are nucleated, and their eNOS-dependent effects might be different from the effects in mammalian, an anuclear RBCs.

Nitrosation of critical thiols was suggested as one of the possible molecular mechanisms underlying the NO-dependent changes of RBC deformability. Nitrosation of the cytoskeletal protein spectrin was proposed to be involved in the control of RBC deformability determined by LORCA (Grau et al., 2013). Likewise, in another study, treatment with NO donors was suggested as a way “to load” RBCs with nitroso-species, including *S*-nitrosohemoglobin (i.e., NO bound to Cys93 in the beta chain of hemoglobin), increased RBC deformability (again assessed by LORCA), and proposed to improve oxygen delivery to the tissues (Riccio et al., 2015). We here confirmed that treatment with the nitrosothiol CysNO induced intracellular nitrosation of the cytoskeletal protein spectrin, as demonstrated before (Grau et al., 2013). However treatment with CysNO alone did not affect RBC deformability or blood viscosity. Instead, we observed that treatment using CysNO protected RBCs against *t*-BuOOH-induced impairment of RBC deformability Similarly Barodka *et al*. and Belanger *et al*. showed a protective effect of sodium nitroprusside on RBC deformability in response to Ca^2+^-stress induced by treatment with Ca^2+^/Ca ionophore (Barodka et al., 2014b; Belanger et al., 2015).

Taking into consideration the well-known effects of oxidants and thiol reactive molecules such as *N*-methylmaleimide and glutaraldehyde on RBC deformability (Fischer et al., 1978; Haest et al., 1980), it is tempting to speculate that nitrosation of critical thiols by CysNO or sodium nitroprusside may protect intracellular proteins from oxidation and/or cross-linking reactions. Other mechanisms by which CysNO may preserve RBC deformability are also possible. It was proposed that nitrosylation of hemoglobin (i.e., formation of a NO-Hb complex centered on the heme iron) may protect against toxicity of *t*-BuOOH in the K562 erythroid cell line, as assessed by EPR (Gorbunov et al., 1997); the authors of this work proposed that the nitrosylated-heme complex is more difficult to oxidize by *t*-BuOOH. Formation of methemoglobin (Trotta et al., 1983) was also proposed to protect RBCs from *t*-BuOOH-induced oxidative degradation of Hb and lipid peroxidation. Another possibility is that treatment of RBCs with nitrosating agents loads RBCs with both high molecular weight and low molecular weight nitroso-species, allowing a more efficient detoxification of the oxidant. Another conclusion based on these findings may be that RBCs from eNOS KO mice might be more susceptible to oxidative stress, as they lack eNOS-derived NO formation. However, it is important to point out that the expression levels of eNOS and the amount of NO released in RBCs are very low as measured and discussed by us (Cortese-Krott et al., 2012b; Cortese-Krott and Kelm, 2014). In general, it is important to take into consideration that the current experimental data do neither allow us to explain the biochemistry nor the molecular mechanisms underlying the effects of oxidants and the protective effects of nitrosothiols or sodium nitroprusside on shear-induced elongation. Therefore, these considerations should be seen as speculative.

### What are the mechanisms responsible for decrease of RBC deformability in hypertensive patients?

This study started with the observation that RBCs from patients with hypertension show decreased RBC deformability and concomitant increase in ROS, while their NO levels in RBCs were unchanged. According to the findings presented here, changes in ROS levels as well as EI in RBCs from hypertensive patients may reflect oxidative modifications of intracellular proteins (hemoglobin, spectrin), which should be investigated in future studies.

Endothelial dysfunction of hypertensive patients (demonstrated here by a decrease in FMD) is not reflected by decreases of NO levels (DAF-FM) or ${\text{NO}}_{2}^{-}$/${\text{NO}}_{3}^{-}$ levels in RBCs, nor does it correlate with RBC deformability. Similarly, in eNOS KO mice, which have increased blood pressure and decreased NO bioavailability (Godecke et al., 1998), RBC deformability is fully preserved. This indicates that decreased NO bioavailability of RBCs or hypertensive state are not major determinants of RBC deformability, but rather the levels of ROS and the intracellular redox state impact RBC deformability.

Although the findings presented in this study clearly indicate that increases in ROS in RBCs seems to be a plausible cause of loss in RBC deformability, as observed in hypertensive patients, there are some important limitations of this study that need to be considered. Hypertension is a complex disease with many influencing factors. For example, patients in our study were smokers or had a history of smoking (1 smoker and 4 ex-smoking individuals of 9 hypertensive individuals; compared to 1 smoker and 3 ex-smoking individuals of 11 normotensive controls). Smoking may increase systemic oxidative stress. In the cohorts, we did not collect information on systemic redox state (levels of thiols, oxidant and antioxidant levels in plasma), such changes may lead to the changes of redox state observed in RBCs from hypertensive patients and therefore is not a cause of hypertensive disease.

Taken together, translational and clinical studies should be focused on understanding the molecular mechanisms and the pathophysiological consequences of decreased RBC deformability in hypertension.

### Summary and future directions

RBCs were considered for a long time as simple “bags” packed with hemoglobin circulating in the blood for the only purposes of gas exchange and maintenance of acid/base equilibria; however evidence is now accumulating that RBC function is far more complex and highly regulated. Importantly, in contrast to the common knowledge that NO directly affects RBC deformability, this study shows that neither eNOS-dependent NO formation nor NO donors affected RBC deformability *per se*; instead, we found that treatment with nitrosothiols contributes to preserve their resilience to intracellular oxidative modifications and loss of membrane flexibility.

More insights on the chemical, biochemical, and biophysical interactions regulating the mechanical properties of RBCs (which include intracellular viscosity largely dependent on Hb, elasticity and plasticity of the cytoskeletal network regulated by protein-protein interactions, and the fluidity of the membrane) will shed further light on the molecular mechanisms of these effects.

Such future studies should include experimental observations of *ex vivo* and *in vivo* behavior of RBCs, experimental identification and verification of specific redox switches involved therein, as well as computer-aided multiscale modeling approaches of RBCs (from the atomistic level of RBC proteins to RBC rheological behavior in flow conditions along the vascular tree), accompanied by studies analyzing the role of redox changes in tissue perfusion *in vivo*. These integrative studies will hopefully provide a link between cell elastic behavior, its physiological role *in vivo* and significances of changes in RBC deformability observed in hypertension and other pathologies.

## Author contributions

LD planned and executed experiments, analyzed data, and drafted the manuscript. TS provided conceptual and intellectual input, drafted, and critically revised the manuscript. RS coordinated the human study, recruited patients, collected blood samples, measured FMD, and critically revised the manuscript; TK, FB, TRS, CK, and WL planned and executed experiments and revised the manuscript. BEI, HG, MF, and MK made substantial contribution in interpretation of the work, and critically revised the work. MC-K designed and coordinated the work and wrote the paper. All authors have given their final approval of the version of the manuscript to be published.

### Conflict of interest statement

The authors declare that the research was conducted in the absence of any commercial or financial relationships that could be construed as a potential conflict of interest.
