# Supplementary material for: On the Effects of Reactive Oxygen Species and Nitric Oxide on Red Blood Cell Deformability
Source: Front Physiol. 2018 May 11;9:332. doi: 10.3389/fphys.2018.00332 (PMC5958211; doi:10.3389/fphys.2018.00332)
Supplement: Supplementary file 1 [file Table_1.docx]

Supplementary Material

On the effects of reactive oxygen species and nitric oxide on red blood cell deformability

Lukas Diederich^1,2^, Tatsiana Suvorava^1,2^, Roberto Sansone^1,2^, TC Stevenson Keller IV ^1,2,4^, Frederik Barbarino^1,2^, Thomas R. Sutton^3^, Christian M. Kramer^1,2^, Wiebke Lückstädt^1,2^, Brant E. Isakson^2,4^, Holger Gohlke^5^, Martin Feelisch^3^, Malte Kelm^1,2^, Miriam M. Cortese-Krott^1,2*^

^1^Cardiovascular Research Laboratory, Division of Cardiology, Pneumology and Vascular Medicine, Medical Faculty, Heinrich-Heine-University Düsseldorf, Düsseldorf, Germany,

^2^CARID, Cardiovascular Research Institute Düsseldorf, Medical Faculty, Heinrich-Heine-University Düsseldorf, Düsseldorf, Germany,

^3^Clinical & Experimental Sciences, Faculty of Medicine, University of Southampton, Southampton, United Kingdom,

^4^Robert M. Berne Cardiovascular Research Center, Department of Molecular Physiology and Biophysics Physics, University of Virginia, Charlottesville, VA, The United States of America,

^5^Institute of Pharmaceutical and Medicinal Chemistry, Heinrich-Heine-University Düsseldorf, Düsseldorf, Germany

*** Correspondence:**Miriam M. Cortese-Krott, PhD,
E-mail: miriam.cortese@uni-duesseldorf.de

Division of Cardiology, Pneumology, and Vascular Medicine,
Medical Faculty, Heinrich -Heine -University of Düsseldorf,
40225 Düsseldorf, Moorenstr. 5, Germany.
Tel. +49 (0) 211 81 15115

# 1. Supplementary material and methods

## Human study

*Cohort study:* All study participants provided written informed consent to study participation before enrollment. The study participants were all males and between 40-60 years old. Study population participants were separated into a hypertensive and healthy normotensive group after measuring 24 hour blood pressure. Patients’ characteristics are provided in the supplementary information table S1. High blood pressure was defined as systolic blood pressure > 135 mm Hg and diastolic blood pressure > 85 mm Hg. For the investigation, all patients were asked to refrain from smoking and remain fasted from the night before (20:00 pm). Current medication was discontinued for at least 1 day before the study.

## Immunoprecipitation

For immunoprecipitation, fresh human blood was collected from the antecubital vein. Red blood cells (RBCs) were separated from citrate-treated whole blood using centrifugation at 800 g for 15 minutes at room temperature (RT). To yield pure RBCs, plasma and buffy coat were removed by aspiration, and the remaining RBC pellets (1 mL) were lysed with toluene. Sample protein concentration was determined after Lowry (DC Protein Assay, Bio-Rad). Samples containing 100 µg/µL proteins were incubated with a mouse anti-human NOS3 antibody (40µg, BD Bioscience) for 1 hour at RT. Addition of protein G Dynabeads Invitrogen) was conducted according to the manufacturer’s instructions. Samples were loaded onto 7% NuPAGE Novex Tris/Acetate precast gels Invitrogen), and western blot was performed using rabbit anti-eNOS antiserum (1:1000, BD Bioscience) and as a secondary antibody goat anti-rabbit antibody conjugated to horse radish peroxidase (1:5000, Rockland) as previously described (Cortese et al., 2008).

## Measurement of hemolysis

Incubation of whole blood was executed as described in chapter §2.8 in the main script. To isolate the supernatant, samples were centrifuged (800 g, 4 °C, 10 minutes). Using a FLUOstar Omega plate reader (BMG Labtech) UV-Vis spectra were measured to determine extracellular hemoglobin released by hemolytic processes. Δabs values were calculated by subtraction of background absorption at 700 nm from absorption at 570 nm (Keller et al., 2017).

# 2. Supplementary Figures and Tables

**2.1 Supplementary Figures**





**Supplementary Figure S1.** RBCs from wildtype (WT) mice (n = 3) do not show any differences in deformability measured by LORCA as compared to RBC from WT mice treated with the nitric oxide synthase (NOS) inhibitor S-ethyl isothiourea (ETU) with a dose of 1.3 µmol/kg/min for 30 minutes (n = 3). In the left panel, the elongation (EI) index against shear stress curve demonstrated no difference between WT mice and mice treated with the NOS inhibitor. 2-way ANOVA, p = 0.0917. In the right panel, the same data are displayed following transformation according to Lineweaver and Burke, only being significant for the highest value of the reciprocal shear stress value corresponding for the lowest EI at the lowest shear stress of 0.30 Pa. 2-way ANOVA, p = 0.0058, Sidak’s test, ****: p < 0.0001.


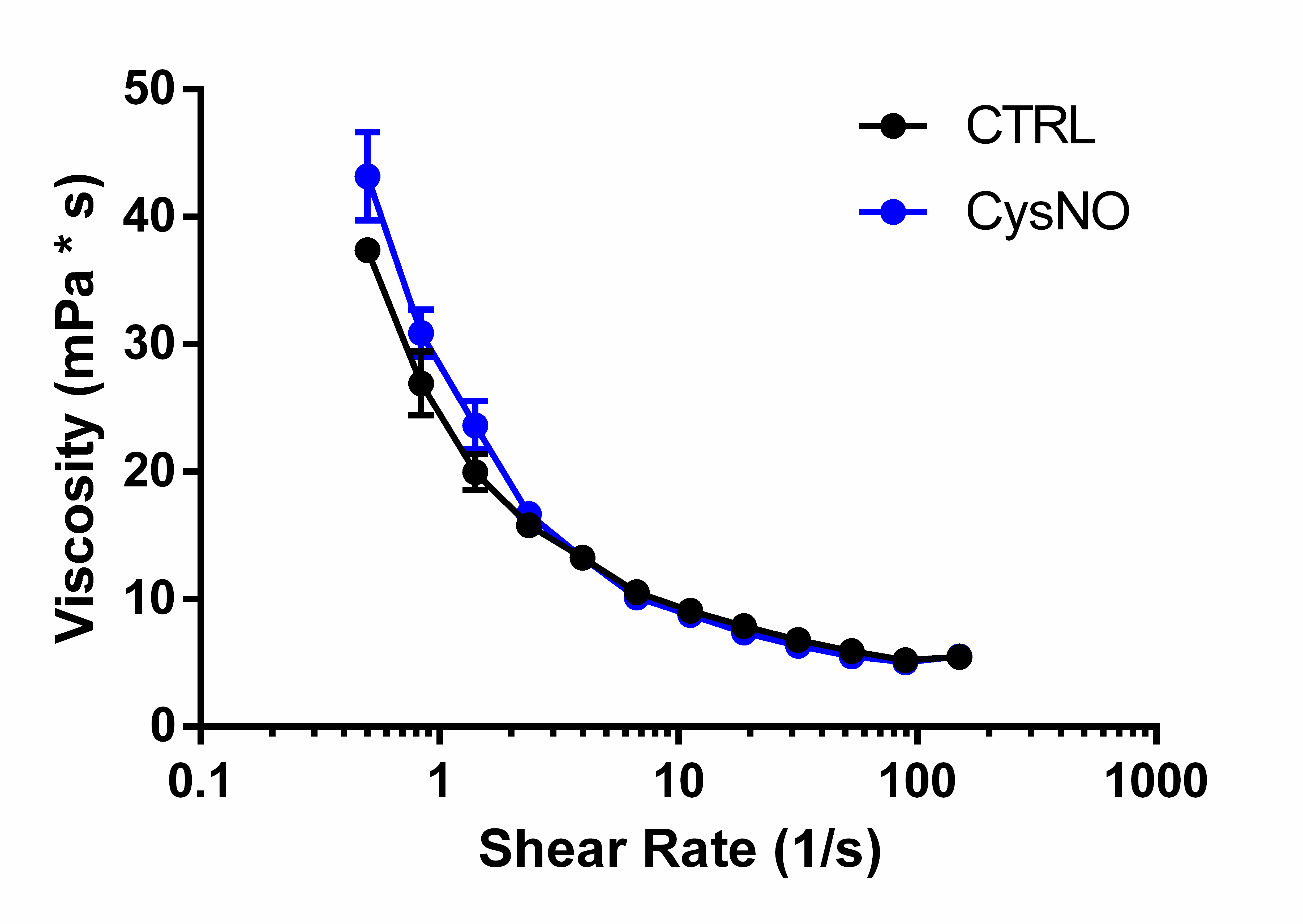


**Supplementary Figure S2.** Whole blood viscosity was not changed upon incubation with 200 µM S-nitrosated cysteine (CysNO). Whole blood was incubated with CysNO and viscosity measured using the LS300. No decrease of viscosity was observed (n = 2). 2-way ANOVA, p = 0.0637.

**

**

**Supplementary Figure S3.** Measurement of hemolysis in whole blood samples (n = 5) as described in §2.8. Δabs was calculated by subtraction of background absorption at 700 nm from that of 570 nm. Incubation of samples with 100 µL H_2_O instead of 100 µL in HBSS^+^ buffered *t*-BuOOH served as positive control for hemolysis. For control conditions (CTRL) samples were incubated with 100 µL HBSS^+^. *1-way RM ANOVA, p = 0.0041 and Dunett’s test vs CTRL*: *p* < 0.05.

**

**

**Supplementary Figure S4.** Whole western blot of Fig. 5 A in the main manuscript. Before membrane blocking and incubation with antibodies, the membrane was cut at 110 kDa using the Novex Sharp Pre-stained protein standard for orientation. Both images were taken from the same membrane. Each lane is labeled with the concentration CysNO used in the treatment of the RBC suspension. (A) Imaging of the anti-mouse Cy3 signal targeted against the mouse anti-spectrin antibody. (B) Imaging of the Cy5-streptavidin coupled antibody targeted against the biotin labeled nitrosothiol-sites.

**2.2 Supplementary Tables**

**Supplementary Table S1.** Human study characteristics**.** The study cohort consisted of male, age-matched, healthy normotensive control subjects (n = 11) and hypertensive patients (n = 9). The three measured parameters for blood pressure were significantly increased while no increases in heart rate were observed. Significant difference was observed in body mass index (BMI), but no differences in glomerular filtration rate, total cholesterol, glycated hemoglobin (HbA1c), plasma glucose, and hemoglobin content were observed. Response in flow-mediated dilation (FMD) was significantly decreased in hypertensive patients, and endothelium-independent dilation using glyceryl trinitrate (GTN) showed a trend to higher reactivity in hypertensive patients. Further characterization of RBCs displayed no changes in eNOS expression, DAF-FM ΔMFI – a nitric oxide sensitive probe - and, Mitosox ΔMFI – a superoxide anion sensitive probe - and Thiol Tracker ΔMFI – a probe with fluorescence dependent on intracellular free thiol groups. At the bottom of the table information regarding current medication is listed. Statistics: Mann-Whitney-U test.

|  | **Healthy subjects** | **Hypertonic subjects** | **p value** |
| --- | --- | --- | --- |
| n(M/F) | 11/0 | 9/0 | - |
| Age [years] | 47.8 ± 5.5 | 50.6 ± 6.9 | 0.38 |
| Systolic blood pressure [mmHg] | 122.7 ± 6.7 | 144.7 ± 6.4 | ˂ 0.0001 |
| Diastolic blood pressure [mmHg] | 76.7 ± 4.9 | 91.9 ± 6.0 | ˂ 0.0001 |
| Mean arterial pressure [mmHg] | 92.0 ± 4.9 | 109.5 ± 5.0 | ˂ 0.0001 |
| Heart rate [1/min] | 65.5 ± 8.6 | 64.0 ± 6.1 | 0.75 |
| Ex Smoking individuals [%] | 27.3 | 44.4 | - |
| Smoking individuals [%] | 9.1 | 11.1 | - |
| Packyears | 10.0 ± 18.3 | 12.2 ± 12.3 | 0.36 |
| BMI [kg/m2] | 26.9 ± 4.2 | 29.6 ± 2.4 | 0.03 |
| GFR [ml/min] | 91.2 ± 11.9 | 88.8 ± 17.4 | 0.70 |
| Total Cholesterol [mg/dL] | 210.5 ± 40.2 | 227.7 ± 38.7 | 0.70 |
| HbA1c [%] | 5.5 ± 0.5 | 5.4 ± 0.3 | 0.61 |
| Plasma glucose [g/dl] | 94.2 ± 5.9 | 92.8 ± 7.6 | 0.67 |
| Hemoglobin [g/dl] | 15.2 ± 0.8 | 15.0 ± 1.0 | 0.84 |
|  |  |  |  |
| Baseline vessel size [mm] | 5.01± 0.69 | 4.76± 0.49 |  |
| FMD [%] | 6.84 ± 0.66 | 5.12± 0.48 | ˂ 0.0001 |
| GTN induced dilatation [%] | 11.3± 0.7 | 12.09± 1.32 | 0.15 |
| Relative RBC eNOS expression | 1.00±0.32 | 1.55±1.20 | 0.35 |
| DAF-FM ΔMFI in RBCs | 659.7±216.5 | 697.1±274.1 | 0.80 |
| Mitosox ΔMFI in RBCs | 4.00±2.83 | 5.56±7.25 | 0.53 |
| Thiol Tracker ΔMFI in RBCs | 67790±5744 | 71294±18123 | 0.74 |
| Nitrite [nM] | 8.67±1.39 | 12.71±2.21 | 0.13 |
| **Medication** |  |  |  |
| CSE-Hemmer [%] | 0.2 | 0.3 |  |
| Simvastatin [mg] | 3.0 | 13.4 |  |
| Beta-blockers [%] | 0.2 | 0.1 |  |
| Beta-blockers (Belok) [mg] | 8.7 | 15.0 |  |
| ACE/AT1 [%] | 0.1 | 0.6 |  |
| ACE/AT1 [mg] | 0.9 | 2.4 |  |

**Supplementary Table S2.** Human RBCs’ characteristics as measured by LORCA. EI at discrete shear stresses (1.73 Pa, 2.68 Pa, and 10.00 Pa) and coefficient of determination, EI_max_, and SS_1/2_ were calculated using a Lineweaver-Burke transformation of human study data (Fig. 1 A). Statistics: Mann-Whitney-U test.

|  | EI at 1.73 Pa | EI at 2.68 Pa | EI at 10.00 Pa | r^2^ Lineweaver-Burke transformation | EI _max_ | SS_1/2_ |
| --- | --- | --- | --- | --- | --- | --- |
| healthy | 0.275±0.022 | 0.354±0.020 | 0.530±0.009 | 0.911 | 0.759±0.152 | 3.659±1.429 |
| hypertension | 0.257±0.007 | 0.337±0.006 | 0.528±0.009 | 0.829 | 0.670±0.089 | 3.257±1.090 |
| *p* - value | 0.06 | 0.04 | 0.73 | - | 0.49 | 0.58 |

**Supplementary Table S3.** Deformability of human RBCs after incubation with *t*-BuOOH as measured by LORCA. EI at discrete shear stresses (1.69 Pa, 3.00 Pa, and 9.48 Pa) and coefficient of determination, EI_max_, and SS_1/2_ were calculated using a Lineweaver-Burke transformation (Fig. 2 C).

| concentration *t*-BuOOH | EI at 1.69 Pa | EI at 3.00 Pa | EI at 9.48 Pa | r^2^ Lineweaver-Burke transformation | EI _max_ | SS_1/2_ |
| --- | --- | --- | --- | --- | --- | --- |
| 0 mM | 0.329±0.023 | 0.423±0.023 | 0.551±0.012 | 0.929 | 0.702±0.065 | 2.122±0.725 |
| 3 mM | 0.276±0.035 | 0.377±0.034 | 0.528±0.018 | 0.876 | 1.126±0.600 | 6.927±5.822 |
| 5 mM | 0.223±0.039 | 0.324±0.041 | 0.491±0.030 | 0.795 | 0.271±1.722 | 5.225±16.960 |
| 7 mM | 0.137±0.059 | 0.220±0.071 | 0.383±0.075 | 0.612 | 0.527±0.541 | 4.223±5.356 |

**Supplementary Table S4.** RBC characteristics of Nrf2 KO and WT mice after incubation with different concentrations of *t*-BuOOH as measured by LORCA. EI at discrete shear stresses (1.69 Pa, 3.00 Pa, and 9.48 Pa) and coefficient of determination, EI_max_, and SS_1/2_ were calculated using a Lineweaver-Burke transformation (Fig. 3 B-D).

|  | concentration *t*-BuOOH | EI at 1.69 Pa | EI at 3.00 Pa | EI at 9.48 Pa | r^2^ Lineweaver-Burke transformation | EI _max_ | SS_1/2_ |
| --- | --- | --- | --- | --- | --- | --- | --- |
| WT | 0 µM | 0.434±0.012 | 0.490±0.019 | 0.535±0.004 | 0.872 | 0.566±0.022 | 1.770±0.069 |
|  | 50 µM | 0.397±0.024 | 0.443±0.016 | 0.493±0.019 | 0.874 | 0.501±0.020 | 0.362±0.100 |
|  | 100 µM | 0.442±0.146 | 0.4633±0.130 | 0.486±0.106 | 0.060 | 0.483±0.116 | 0.079±0.119 |
| NRF2 KO | 0 µM | 0.437±0.012 | 0.491±0.020 | 0.527±0.011 | 0.8021 | 0.567±0.036 | 1.769±0.114 |
|  | 50 µM | 0.362±0.014 | 0.410±0.027 | 0.456±0.046 | 0.8663 | 0.469±0.047 | 0.471±0.172 |
|  | 100 µM | 0.318±0.110 | 0.352±0.096 | 0.386±0.072 | 0.1797 | 0.376±0.083 | 0.162±0.129 |

**Supplementary Table S5.** Human RBC deformability after incubation with different concentrations of DEA/NO as measured by LORCA. EI at discrete shear stresses (1.69 Pa, 3.00 Pa, and 9.48 Pa) and coefficient of determination, EI_max_, and SS_1/2_ were calculated using a Lineweaver-Burke transformation (Fig. 4 A-B).

| concentration DEA/NO | EI at 1.69 Pa | EI at 3.00 Pa | EI at 9.48 Pa | r^2^ Lineweaver-Burke transformation | EI _max_ | SS_1/2_ |
| --- | --- | --- | --- | --- | --- | --- |
| 0 µM | 0.412±0.014 | 0.484±0.010 | 0.567±0.007 | 0.985 | 0.635±0.016 | 1.017±0.083 |
| 1 µM | 0.417±0.014 | 0.488±0.009 | 0.568±0.006 | 0.976 | 0.655±0.019 | 1.129±0.175 |
| 10 µM | 0.411±0.011 | 0.484±0.008 | 0.567±0.006 | 0.951 | 0.641±0.023 | 1.056±0.246 |
| 100 µM | 0.406±0.011 | 0.481±0.007 | 0.565±0.005 | 0.969 | 0.635±0.014 | 1.055±0.180 |
| 200 µM | 0.395±0.016 | 0.473±0.010 | 0.561±0.006 | 0.922 | 0.667±0.048 | 1.184±0.092 |

**Supplementary Table S6.** RBC characteristics of eNOS KO and WT measured by LORCA. EI at discrete shear stresses (1.69 Pa, 3.00 Pa, and 9.48 Pa) and coefficient of determination, EI_max_, and SS_1/2_ were calculated using a Lineweaver-Burke transformation (Fig. 4 C-D).

|  | |  |  | | |  |  | | |  | |  |
| --- | --- | --- | --- | --- | --- | --- | --- | --- | --- | --- | --- | --- |
|  | EI at 1.69 Pa | | | EI at 3.00 Pa | EI at 9.48 Pa | | | r^2^ Lineweaver-Burke transformation | EI _max_ | | SS_1/2_ | |
| WT | 0.423±0.006 | | | 0.473±0.004 | 0.538±0.002 | | | 0.960 | 0.547±0.004 | | 0.391±0.046 | |
| eNOS KO | 0.421±0.004 | | | 0.472±0.004 | 0.539±0.003 | | | 0.951 | 0.546±0.004 | | 0.376±0.041 | |

**Supplementary Table S7.** Changes of human RBC deformability after incubation with CysNO alone and CysNO before/after *t*-BuOOH treatment as measured by LORCA. EI at discrete shear stresses (1.69 Pa, 3.00 Pa, and 9.48 Pa) and coefficient of determination, EI_max_, and SS_1/2_ calculated using a Lineweaver-Burke transformation (Fig. 5 B-D).

| Concentration | | E_i_ at 1.69 Pa | E_i_ at 3.00 Pa | E_i_ at 9.48 Pa | r^2^ lineweaver-burke | E_i max_ | SS_1/2_ |
| --- | --- | --- | --- | --- | --- | --- | --- |
| 0 µM CysNO | | 0.321±0.008 | 0.416±0.004 | 0.550±0.005 | 0.942 | 0.653±0.070 | 1.776±0.638 |
| 1 nM CysNO | | 0.334±0.018 | 0.421±0.012 | 0.550±0.005 | 0.859 | 0.598±0.063 | 1.242±0.607 |
| 1 µM CysNO | | 0.332±0.011 | 0.420±0.008 | 0.547±0.007 | 0.873 | 0.573±0.051 | 1.047±0.474 |
| 1 mM CysNO | | 0.312±0.0186 | 0.409±0.011 | 0.546±0.012 | 0.921 | 0.674±0.077 | 2.103±0.747 |
| 50 mM CysNO | | 0.216±0.078 | 0.301±0.098 | 0.442±0.113 | 0.626 | 0.509±0.276 | 1.972±1.641 |
| control | | 0.337±0.012 | 0.429±0.010 | 0.556±0.006 | 0.913 | 0.668±0.075 | 1.720±0.628 |
| 3mM *t*-BuOOH | | 0.125±0.054 | 0.135±0.080 | 0.181±0.138 | 0.024 | 0.183±0.125 | 0.354±0.632 |
| 1 nM CysNO | 3 mM *t*-BuOOH | 0.132±0.056 | 0.160±0.080 | 0.237±0.122 | 0.278 | 0.246±0.142 | 0.695±0.813 |
| 1 µM CysNO | 3 mM *t*-BuOOH | 0.184±0.130 | 0.220±0.164 | 0.294±0.210 | 0.185 | 0.300±0.198 | 0.658±0.514 |
| 1 mM CysNO | 3 mM *t*-BuOOH | 0.208±0.114 | 0.261±0.132 | 0.361±0.151 | 0.412 | 0.359±0.144 | 0.980±0.812 |
| 50 mM CysNO | 3 mM *t*-BuOOH | 0.090±0.024 | 0.112±0.048 | 0.177±0.102 | 0.158 | 0.169±0.105 | 0.792±1.269 |
| 3 mM *t*-BuOOH | 1 nM CysNO | 0.123±0.073 | 0.149±0.089 | 0.212±0.123 | 0.235 | 0.209±0.120 | 0.572±0.625 |
| 3 mM *t*-BuOOH | 1 µM CysNO | 0.169±0.092 | 0.214±0.122 | 0.300±0.177 | 0.306 | 0.310±0.231 | 1.020±1.137 |
| 3 mM *t*-BuOOH | 1 mM CysNO | 0.172±0.041 | 0.216±0.070 | 0.310±0.103 | 0.554 | 0.331±0.153 | 1.120±1.183 |
| 3 mM *t*-BuOOH | 50 mM CysNO | 0.162±0.077 | 0.191±0.127 | 0.240±0.201 | 0.040 | 0.305±0.327 | 1.055±1.785 |

## Supplementary Table S8. Methodological details and main findings of the published studies on the effects of NO on RBC deformability are listed.

| Protocol | (Bor-Kucukatay et al., 2003) | (Grau et al., 2013) | (Riccio et al., 2015) | (Belanger et al., 2015) |
| --- | --- | --- | --- | --- |
| sample source | whole blood washed with PBS (pH=7.4) | whole blood centrifuged and cell pellet re-suspended | stored RBCs | whole blood |
| treatment buffer | plasma | autologous plasma | PBS  (pH = 7.4) | heparinized whole blood |
| timeframe | 4-6 hours after collection | - | - | - |
| treatment hct | 40% | 40% | 35% | - |
| treatment | 1 µM-0.01 M  L-NAME & SMT,  0.1 µM- 0.1 mM SNP & DETA-NONOate | 3 mM L-arginine,  10 µM L-NIO,  10 µM wortmannin, 200 pM insulin,  300 µM cPTIO,  100 µM SNP | deoxygenated DEA/NO, PROLI/NO 1:250; 1:62.5; 1:25  (NO: Hb ratio) | 10 µM SNP,  10 µM DEA-NONOate,  1 mM L-NAME,  3 mM L-Arginine |
| treatment duration | 60 minutes at room temperature | 60 minutes at 37°C | 15 minutes | 60 minutes at room temperature |
| rheometer medium | 70 kDA dextran | isotonic PVP | isotonic PVP | 20 mPa*s carrier solution pH = 7.4 on a PVP base |
| final hct | low hct solution | 0.4% | 0.21% | 3.75% of whole blood |
| shear rate (1/s) | 0.5 - 15 Pa* | 10 - 1666.67 | 3 and 30 Pa* | 800 |
| finding | NO is a regulator of RBC deformability | EImax increased with L-arginine, insulin, SNP  EImax decreased with L-NIO, wortmannin, cPTIO | DEA/NO PROLI/NO are able to improve deformability | no changes of deformability for any treatment under basal conditions |
|  |  |  |  |  |
| * no viscosity of measuring medium available, hct- hematocrit, PVP- polyvinylpyrrolidone, PBS – phosphate buffer saline | | | | |

# 3. References

Belanger, A.M., Keggi, C., Kanias, T., Gladwin, M.T., and Kim-Shapiro, D.B. (2015). Effects of nitric oxide and its congeners on sickle red blood cell deformability. *Transfusion* 55**,** 2464-2472.

Bor-Kucukatay, M., Wenby, R.B., Meiselman, H.J., and Baskurt, O.K. (2003). Effects of nitric oxide on red blood cell deformability. *Am J Physiol Heart Circ Physiol* 284**,** H1577-1584.

Cortese, M.M., Suschek, C.V., Wetzel, W., Kroncke, K.D., and Kolb-Bachofen, V. (2008). Zinc protects endothelial cells from hydrogen peroxide via Nrf2-dependent stimulation of glutathione biosynthesis. *Free Radic Biol Med* 44**,** 2002-2012.

Grau, M., Pauly, S., Ali, J., Walpurgis, K., Thevis, M., Bloch, W., and Suhr, F. (2013). RBC-NOS-dependent S-nitrosylation of cytoskeletal proteins improves RBC deformability. *PLoS One* 8**,** e56759.

Keller, A.S., Diederich, L., Panknin, C., Delalio, L.J., Drake, J.C., Sherman, R., Jackson, E.K., Yan, Z., Kelm, M., Cortese-Krott, M.M., and Isakson, B.E. (2017). Possible roles for ATP release from RBCs exclude the cAMP-mediated Panx1 pathway. *Am J Physiol Cell Physiol***,** ajpcell 00178 02017.

Riccio, D.A., Zhu, H., Foster, M.W., Huang, B., Hofmann, C.L., Palmer, G.M., and Mcmahon, T.J. (2015). Renitrosylation of banked human red blood cells improves deformability and reduces adhesivity. *Transfusion* 55**,** 2452-2463.
